# Quantitative Physiologic MRI Combined with Feature Engineering for Developing Machine Learning-Based Prediction Models to Distinguish Glioblastomas from Single Brain Metastases

**DOI:** 10.3390/diagnostics15010038

**Published:** 2024-12-27

**Authors:** Seyyed Ali Hosseini, Stijn Servaes, Brandon Hall, Sourav Bhaduri, Archith Rajan, Pedro Rosa-Neto, Steven Brem, Laurie A. Loevner, Suyash Mohan, Sanjeev Chawla

**Affiliations:** 1 Translational Neuroimaging Laboratory, The McGill University Research Centre for Studies in Aging, Douglas Hospital, McGill University, Montreal, QC H4H 1R3, Canada; 2 Department of Neurology and Neurosurgery, Faculty of Medicine, McGill University, Montreal, QC H3A 2B4, Canada; 3 Institute for Advancing Intelligence (IAI), The Chatterjee Group—Centre for Research and Education in Science and Technology (TCG CREST), Kolkata 700091, India; 4 Academy of Scientific and Innovative Research (AcSIR), Ghaziabad 201002, India; 5 Department of Radiology, Perelman School of Medicine, University of Pennsylvania, Philadelphia, PA 19104, USA; 6 Department of Neurosurgery, Perelman School of Medicine, University of Pennsylvania, Philadelphia, PA 19104, USA

**Keywords:** glioblastomas, brain metastases, MRI, machine learning, feature engineering

## Abstract

**Background**: The accurate and early distinction of glioblastomas (GBMs) from single brain metastases (BMs) provides a window of opportunity for reframing treatment strategies enabling optimal and timely therapeutic interventions. We sought to leverage physiologically sensitive parameters derived from diffusion tensor imaging (DTI) and dynamic susceptibility contrast (DSC)–perfusion-weighted imaging (PWI) along with machine learning-based methods to distinguish GBMs from single BMs. **Methods**: Patients with histopathology-confirmed GBMs (*n* = 62) and BMs (*n* = 26) and exhibiting contrast-enhancing regions (CERs) underwent 3T anatomical imaging, DTI and DSC-PWI prior to treatment. Median values of mean diffusivity (MD), fractional anisotropy, linear, planar and spheric anisotropic coefficients, and relative cerebral blood volume (rCBV) and maximum rCBV values were measured from CERs and immediate peritumor regions. Data normalization and scaling were performed. In the next step, most relevant features were extracted (non-interacting features), which were subsequently used to generate a set of new, innovative, high-order features (interacting features) using a feature engineering method. Finally, 10 machine learning classifiers were employed in distinguishing GBMs and BMs. Cross-validation and receiver operating characteristic (ROC) curve analyses were performed to determine the diagnostic performance. **Results**: A random forest classifier with ANOVA F-value feature selection algorithm using both interacting and non-interacting features provided the best diagnostic performance in distinguishing GBMs from BMs with an area under the ROC curve of 92.67%, a classification accuracy of 87.8%, a sensitivity of 73.64% and a specificity of 97.5%. **Conclusions**: A machine learning based approach involving the combined use of interacting and non-interacting physiological MRI parameters shows promise to differentiate between GBMs and BMs with high accuracy.

## 1. Background

Glioblastomas (GBMs) and single brain metastases (BMs) are the two most common intra-axial brain neoplasms in adults [1]. Accurate preoperative diagnosis is often crucial because the management and prognosis of these two neoplasms are quite different [2,3,4]. While patients with GBMs are almost always treated by maximal or supramaximal surgical resection followed by concurrent chemoradiation therapy, patients with suspected BMs without a clinical history of systemic cancer usually undergo a complete systemic staging to determine the site of primary cancer and status of distant metastases before initiating any treatment (surgical, medical or radiation therapy) [1]. Therefore, the accurate and early diagnosis of GBMs versus single BMs provides a window of opportunity for reframing treatment strategies, enabling optimal and timely therapeutic interventions in these patients.

Conventional magnetic resonance imaging (MRI) such as post-contrast T1-weighted and T2-FLAIR images provide refined anatomic details, including variable degrees of necrosis, microhemorrhage, vasogenic edema, and heterogeneous contrast enhancement within the tumor bed [5]. On conventional neuroimaging, both GBMs and BMs exhibit similar degrees of signal intensity and tissue contrast patterns, thus presenting a considerable diagnostic dilemma to radiologists and oncologists in the accurate differentiation of these two entities [6]. However, the continuous refinements in physiologic MRI techniques such as diffusion tensor imaging (DTI) and dynamic susceptibility contrast–perfusion-weighted imaging (DSC-PWI) have provided new insights into the tumor biology and microenvironment [7]. Previous studies have documented the clinical utility of DTI and DSC-PWI in the differentiation of GBMs and BMs with variable success rates [8,9,10,11,12,13,14]. However, the reported threshold values of a single parameter or a combination of diffusion and perfusion MRI-derived quantitative parameters vary widely across different studies, adversely impacting physicians’ confidence in the reliable utilization of these threshold values in the classification of these two most common neoplasms.

To address this issue, some studies have reported promising findings of machine learning-based radiomic features extracted from anatomical images in distinguishing GBMs and BMs [15,16,17,18,19,20]. However, these studies were focused on using a very large number of shape- and texture-based features (overwhelming volume of data), which are often difficult to manage even after applying dimensionality reduction methods, besides being prone to data overfitting and spurious relationships [21,22]. Moreover, these data-driven radiomic features fail to provide any meaningful physiologically sensitive information and biological interpretation [23], which limit the wider adoption of these machine learning-based prediction models in routine clinical settings. Furthermore, radiomic features are vulnerable to many influential factors [24], including pre- [25] or post-processing [26,27,28,29] steps.

Feature engineering is a novel machine learning-based approach in which raw features are algorithmically transformed into a set of new polynomial features [30,31]. These innovative features can better capture the underlying patterns and non-linear interactions among variables in building more robust and accurate machine learning-based classification models. Previous studies have shown the potential of feature engineering techniques in extracting useful information from unprocessed data and improving the prediction capability of machine learning-based models [32,33]. To the best of our knowledge, this is the first study that has implemented a feature engineering method on quantitative DTI and DSC-PWI image-derived parameters in a neurooncologic application.

The present study was conducted with the hypothesis that physiologically sensitive quantitative parameters computed from DTI and DSC-PWI combined with feature engineering- and machine learning-based techniques will facilitate the distinction of GBMs and BMs with high accuracy.

## 2. Materials and Methods

### 2.1. Patient Population and Clinical Data

This retrospective study was approved by the Institutional Review Board and was compliant with the HIPAA (the Health Insurance Portability and Accountability Act). Data from 88 patients with single contrast-enhanced lesions on post-contrast T1-weighted images were evaluated. Patients with multiple brain lesions, non-enhancing tumors, or a clinical history of receiving any prior therapy were excluded. All patients underwent gross total or near-total resection of the enhanced tumor. The histopathology of the resected tissue confirmed the diagnosis of GBMs in 62 patients and BMs in 26 patients. The demographic characteristics of GBM and BM patients at initial presentation are summarized in Table 1.

### 2.2. Data Acquisition

All patients underwent MRI on a 3T Tim Trio whole-body MR scanner (Siemens, Erlangen, Germany) equipped with a 12-channel phased array head coil. The anatomical imaging protocol included axial 3D-T_1_-weighted magnetization-prepared rapid acquisition of gradient echo (T_1_-MPRAGE) imaging and an axial T_2_-fluid-attenuated inversion recovery (T_2_-FLAIR) imaging using standard parameters. The post-contrast T_1_-weighted images were acquired with the same parameters as the pre-contrast acquisition after the administration of a standard dose (0.14 mmol/kg) of gadolinium-based contrast agent using a power injector (Medrad, Idianola, PA, USA).

### 2.3. Diffusion Tensor Imaging

Axial DTI data were acquired using 30 noncollinear/noncoplanar directions with a single-shot spin-echo, echo-planar read-out sequence with parallel imaging by using generalized autocalibrating partially parallel acquisition (GRAPPA) and an acceleration factor of 2. The sequence parameters were as follows: repetition time (TR)/echo time (TE) = 5000/86 ms, number of excitations (NEX) = 3, field of view (FOV) = 22 × 22 cm^2^, matrix size = 128 × 128, in-plane resolution = 1.72 × 1.72 mm^2^; slice thickness = 3 mm; *b* = 0, 1000 s/mm^2^; number of slices = 40; acquisition time = 8 min.

### 2.4. Dynamic Susceptibility Contrast–Perfusion-Weighted Imaging

For axial DSC-PWI, a bolus of contrast agent was injected with a preloading dose of 0.07 mmol/kg to reduce the effect of contrast agent leakage on CBV measurements. A T_2_*-weighted gradient-echo EPI was used during the second 0.07 mmol/kg bolus of contrast agent for the DSC-PWI. The injection rate was 5 mL/s for all patients and was immediately followed by a flush of saline (total of 20 mL at the same rate). The sequence parameters were as follows: TR/TE = 2000/45 ms; FOV = 22 × 22 cm^2^; matrix size = 128 × 128; in-plane resolution = 1.72 × 1.72 mm^2^; slice thickness = 3 mm; BW = 1346 Hz/pixel; flip angle = 90°; EPI factor = 128; echo spacing = 0.83; acquisition time = 3 min and 10 s. Forty-five sequential measurements were acquired for each section with a temporal resolution of 2.17 s.

### 2.5. Image Processing and Estimation of Quantitative Parameters

The diffusion-weighted images were co-registered to the non-diffusion-weighted (*b* = 0) images by using a 3D affine transformation estimated by maximizing the mutual information between the images using an in-house algorithm (IDL; ITT Visual Information Solutions, Boulder, CO, USA). This step was performed to minimize the artifacts induced by eddy currents and/or subject motion. The corrected raw images were combined to estimate rotationally invariant DTI parameter maps using DTIStudio, Version 3.0 (H. Jiang, S. Mori; Johns Hopkins University, Baltimore, MD, USA). Pixel-wise mean diffusivity (MD), fractional anisotropy (FA), coefficient of linear anisotropy (CL), planar anisotropy (CP), and spherical anisotropy (CS) maps were computed by methods reported previously [9,34]. The DSC-PWI data were processed using NordicICE software 4.1.0. (NordicNeuroLab, Bergen, Norway). Briefly, a well-established tracer kinetic model for the first-pass data was applied to obtain CBV maps. To reduce the effects of recirculation, the gamma-variate function, which is an approximation of the first-pass response as it would appear in the absence of recirculation, was fitted to the 1/T2* curves. Subsequently, dynamic curves were mathematically corrected to reduce contrast agent leakage effects [35,36]. After reducing the effects of recirculation and leakage of the contrast agent, CBV was computed with numeric integration of the curve.

The DTI-derived maps, CBV maps, and T_2_-FLAIR images were resliced and co-registered to post-contrast T_1_-weighted images using a 3D non-rigid transformation and mutual information by combining affine transformation and discrete sine bases up to the 2nd order, depending on the degree of misalignment. As GBMs are extremely heterogenous and infiltrative tumors, a semiautomatic approach was used to segment the lesions into contrast-enhanced regions (CERs) and immediate peritumoral regions (IPRs) by using a signal intensity-based thresholding method [9,34]. Briefly, one region of interest (ROI) was drawn over T2-FLAIR abnormality on every section to create a 3D composite mask. Similarly, another mask was drawn on the post-contrast T1-weighted images for the contralateral normal white matter. The CER was defined as the region with an enhancement higher than the mean + 3 SDs of the signal intensity from the white matter. The IPR was chosen as a 4 mm wide band around the CER. Care was taken to exclude surrounding normal brain vessels. Manual inspections were performed by an experienced, board-certified neuroradiologist to correct for any pixel anomalies present within the segmented regions. Accordingly, these regions of interest (ROIs) were modified manually by adding pixels for tumor regions not included in the initial ROIs or by removing pixels for non-tumor regions included in the initial ROIs. The median values of DTI metrics (MD, FA, CL, CP, and CS) from CERs and IPRs were computed. The CBV values from CERs and IPRs were normalized by the corresponding values from contralateral normal white matter regions to obtain the relative CBV (rCBV). The top 90th percentile rCBV values were also measured from CER and IPR and reported as rCBV_max_. This method allows the reliable estimation of CBV values from the most malignant component of neoplasms analogous to the “hot-spot” method [37].

### 2.6. Machine Learning-Based Analytical Approach

**Figure 1:**
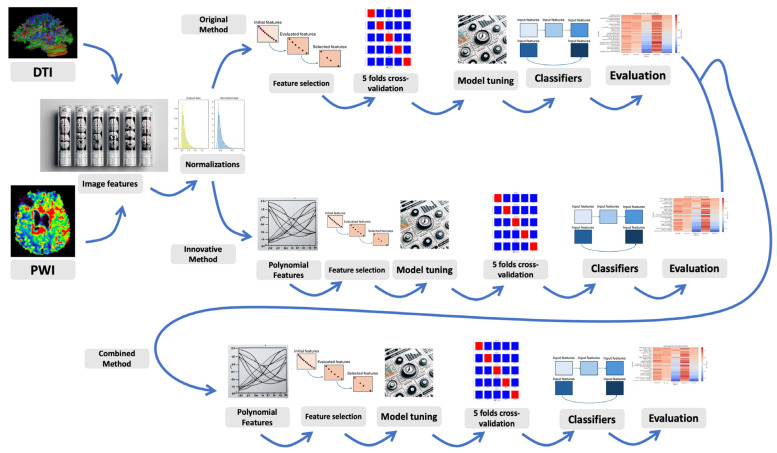
The image processing pipeline provides an overview of the data analytical components including image registration, tissue segmentation, feature selection, model building machine learning-based algorithms, and diagnostic performance metrics.

**Figure 2:**
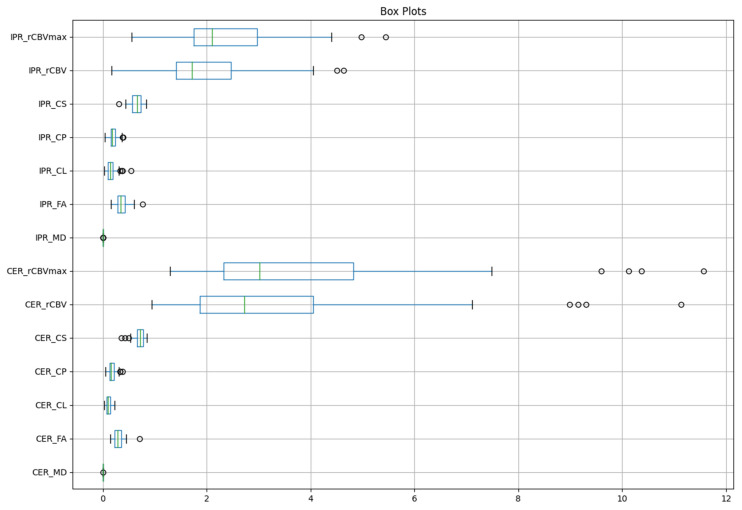
Image data distribution before pre-processing based on minimum, first quartile (Q1), median, third quartile (Q3), and maximum. The box itself represents the interquartile range (IQR), which is the range between the first quartile (25th percentile) and the third quartile (75th percentile). The horizontal line inside the box indicates the median of the data. The “whiskers” extend from the box to the smallest and largest values within 1.5 × IQR from the Q1 and Q3, respectively. Points outside of the whiskers are considered outliers and are represented as individual dots. Abbreviations: contrast-enhanced region (CER), immediate peritumoral region (IPR), mean diffusivity (MD), fractional anisotropy (FA), coefficient of linear anisotropy (CL), planar anisotropy (CP), spherical anisotropy (CS) maps, relative cerebral blood volume (rCBV), and maximum rCBV (rCBV_max_).

The image processing pipeline, as shown in Figure 1, provides an overview of data analytical components including image registration, tissue segmentation, feature selection, model building machine learning-based algorithms, and analyses of diagnostic performance metrics. Figure 2 shows the distribution of imaging data before pre-processing. All data processing was performed using an in-house algorithm implemented on the scikit-learn library in Python 3.9.12.

### 2.7. Data Normalization

All the preprocessing steps were applied after data splitting to mitigate the chance of data leakage. The preprocessing included data normalization to offset any image size and quality variations. This step is important to decrease the impact of outliers and the chances of data overfitting. The data were normalized using a min-max scaler from the Scikit-learn library, which rescaled each input feature individually, and the normalized data varied between the range of 0 and 1 [38].

### 2.8. Data Scaling

Following normalization, data scaling was performed to adjust the scale of the features. While normalization was used to scale the features within a bounded range [0 and 1], scaling was particularly important for our study to manage features with differing units or scales of measurement. This step was vital for algorithms that compute distances between data points, like k-nearest neighbors (KNN) and support vector machines (SVM), as it ensured that the distance metrics did not become skewed due to the different scales of the features.

### 2.9. Feature Selection

It is important to eliminate irrelevant or redundant variables that may cause data overfitting and may bias the performance of the prediction model. Multiple feature selection algorithms, including ANOVA F-value, mutual information, χ^2^, and selection from the models such as logistic regression and random forest were employed to select image features. The data were divided into 2 mutually exclusive training (80%) and testing (20%) sets using the stratified split method.

Three distinct approaches for feature selection were used in the present study.

#### 2.9.1. Conventional/Traditional Method

Initially, we adopted the conventional method for feature representation, utilizing normalized and scaled features without any additional processing step. The primary objective was to establish a baseline for model performance, ensuring that any enhancements achieved through more sophisticated methods could be accurately measured. The simplicity of the original method also facilitated a clearer interpretation of how each feature influenced the model’s predictions, maintaining transparency and clarity in our initial models. Therefore, these features went through feature selection and were followed by classifiers.

#### 2.9.2. Innovative Method

To search for more hidden features and underlying patterns, a feature engineering method was employed. This process involved several key strategies:

**Polynomial Transformations**: We applied polynomial transformations up to the second degree to capture both linear and non-linear relationships between the raw features. This method was applied using the PolynomialFeatures function of the scikit-learn data mining library, whereby the original features were transformed into new features by raising existing features to different powers (like squaring or cubing them). For instance, MD^2^, FA^2^, etc., were created as new features from raw features such as MD and FA.

**Domain-Specific Features**: These features consisted of ratios and interaction terms derived from DTI and DSC-PWI data; for example, features like the ratio of MD to FA and the product of rCBV and FA.

**Feature Selection Process**: Previously, five different feature selection algorithms were used, which pointed to ten very important first-degree terms of the original set. The selected features were then taken through the polynomial transformation process, producing a new set of features, both the initial and interaction term features.

**Quantification of Feature Space Expansion**: The total number of features, m, after polynomial transformation is explained by the following equation:

$$Total\;Number\;of\;Features:\;m=\frac{n(n+1)}{2}$$

For “*n* = 10” original features, the total number of features “*m* = 55”. These 55 features consisted of 10 original features (first-degree terms) and 45 interaction features (second-degree terms).

#### 2.9.3. Combined Method

In recognition of the strengths of both the original and innovative methods, a combined method of feature representation was constructed by integrating these two methods. By merging these approaches, we sought to create a feature set that was not only highly informative and capable of capturing complex patterns but also retained essential characteristics of the data to ensure the robustness and generalizability of the model.

### 2.10. Feature Importance Computation

**Figure 3:**
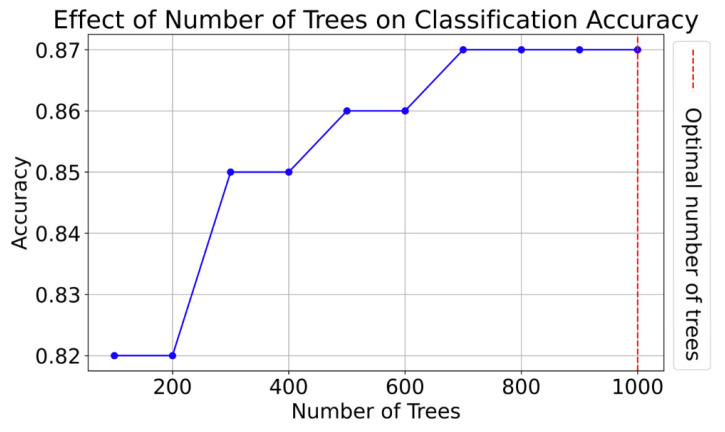
The effect of the number of trees on the classification accuracy between GBMs and BMs.

The feature importance in the training dataset was ranked using a random forest (RF) algorithm, known for its efficiency in handling correlated and redundant features [39]. Initially, an RF with 100 decision trees was embedded in a loop for binary classification (GBMs and BMs). Through systematic testing, the number of trees was incrementally increased and the impact on classification accuracy was observed. After evaluating various configurations, 1000 decision trees were eventually chosen, as further increasing the number did not notably improve classification error (Figure 3). The implementation was carried out using the RandomForestClassifier from scikit-learn, and features scored with higher values indicated greater importance.

### 2.11. Optimization and Performance Evaluation of Prediction Models

Each classifier used in the present study underwent a comprehensive hyperparameter optimization using the Grid Search technique to determine the best hyperparameters for each classifier. Such a rigorous technical process was essential to make sure that each model performed to its optimal capability.

For the optimization of hyperparameters, the Grid Search method was used, which allows for cycling through all the desired combinations for several hyperparameters in one dimension. In each classifier type, certain grids were identified depending on the impact of hyperparameters. For instance, in the case of random forest, the following grid was used for the search: number of trees, 100, 500, 1000, 1500; maximum depth, 10, 20, 30, ‘none’; and minimum sample splitting, 2, 5, 10. The optimization process was intended to maximize AUC-ROC and diagnostic metrics such as accuracy, sensitivity, and specificity.

To estimate the reliability of the prediction models, five-fold cross-validation tests were performed by partitioning the dataset into five folds, iteratively training the model on four folds and validating on the remaining fold. Additionally, receiver operating characteristic (ROC) curve analyses were performed to determine the diagnostic performance of the models. Performance metrics included accuracy, sensitivity, specificity, area under the ROC curve (AUC), and F1 score.

## 3. Results

**Figure 4:**
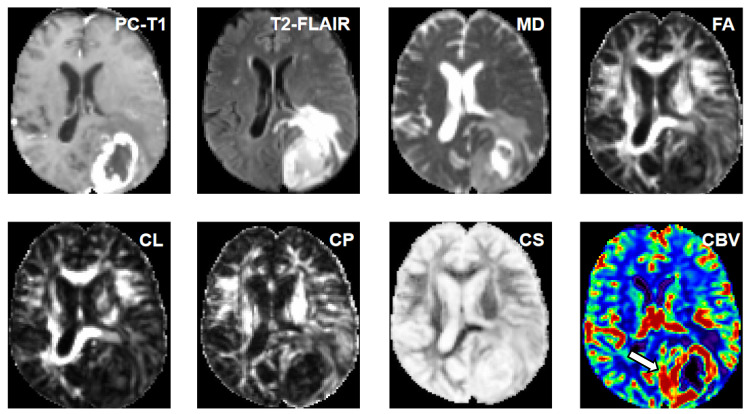
A 65-year-old male patient with a glioblastoma in the left parietal-occipital region. Axial post contrast T1-weighted, and T2-FLAIR images show ring-enhancement and extensive vasogenic edema. DTI derived maps (MD, FA, CL, CP and CS) are shown and CBV map showing high perfusion from CER and IPR of neoplasm indicating neoplastic infiltration (arrow).

**Figure 5:**
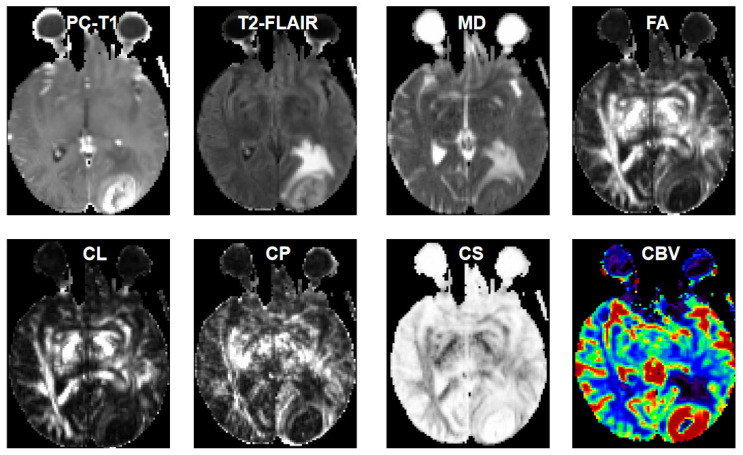
A 51 year old male patient with metastatic lung adenocarcinoma in the left occipital lobe. Axial post contrast T1-weighted, and T2-FLAIR images show ring-enhancement and extensive edema. DTI derived maps (MD, FA, CL, CP and CS) are shown and CBV map showing high perfusion from CER of neoplasm. Please note lower CBV value of this BM from IPR than that of GBM (shown in Figure 4).

**Figure 6:**
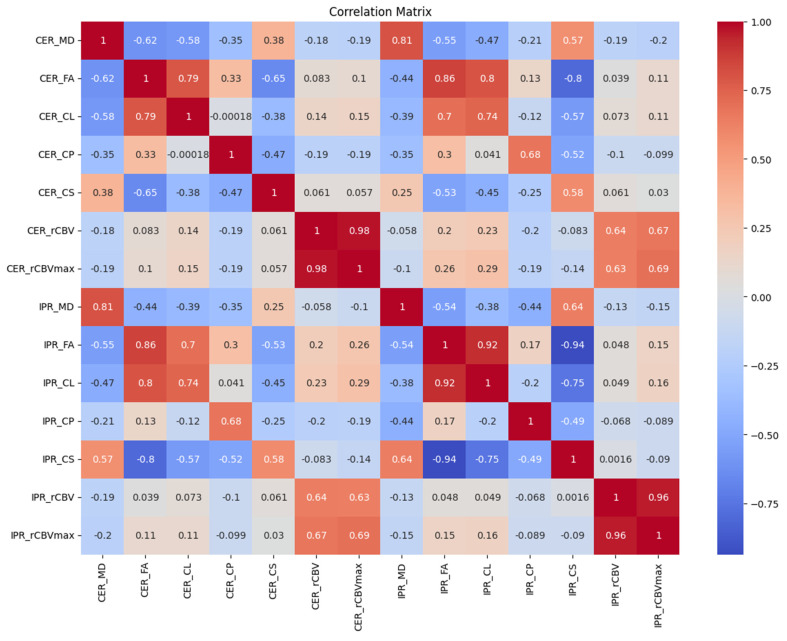
Correlation matrix between various image features extracted in this study. In this matrix: Each cell in the grid represents the value of the correlation coefficient between two variables. The correlation coefficient values range from −1 to 1, where 1 indicates a perfect positive linear relationship, −1 shows a perfect negative linear relationship, and 0 indicates no linear relationship. Abbreviations: contrast-enhanced region (CER), immediate peritumoral region (IPR), mean diffusivity (MD), fractional anisotropy (FA), coefficient of linear anisotropy (CL), planar anisotropy (CP), spherical anisotropy (CS) maps, relative cerebral blood volume (rCBV), and maximum rCBV (rCBV_max_).

Figure 4 and Figure 5 show representative anatomical MR images and quantitative DTI maps and CBV maps from patients with GBMs and BMs, respectively. Figure 6 illustrates the correlation coefficients between pairs of image features extracted in this study.

**Figure 7:**
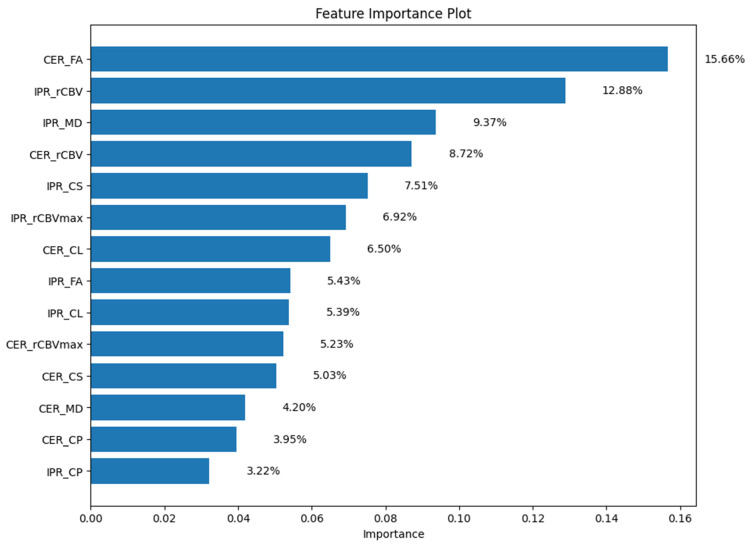
Ranked feature importance based on the random forest classifier are shown. Abbreviations: contrast-enhanced region (CER), immediate peritumoral region (IPR), mean diffusivity (MD), fractional anisotropy (FA), coefficient of linear anisotropy (CL), planar anisotropy (CP), spherical anisotropy (CS) maps, relative cerebral blood volume (rCBV), and maximum rCBV (rCBV_max_).

The random forest-driven feature importance plot is shown in Figure 7. The permutation importance index was used to assess feature significance, as it provides unbiased results across different types of features and is less sensitive to the intrinsic data variability generally observed in complex datasets. Among all independent features, CER_FA (15.66%), IPR_rCBV (12.88%), and IPR_MD (9.37%) had the highest feature scores (indicator of feature relevance) for distinguishing GBMs from BMs.

**Figure 8:**
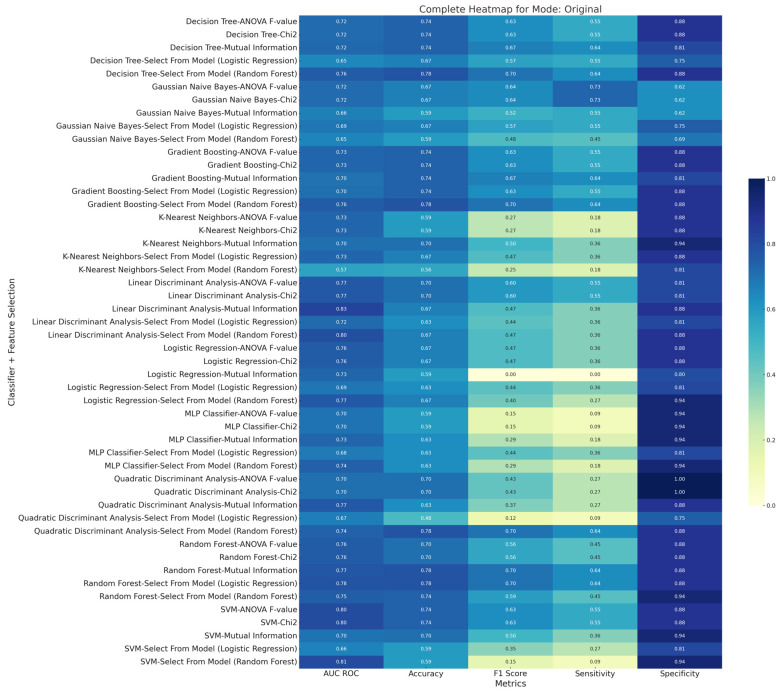
Diagnostic performance metric (AUC ROC, accuracy, sensitivity, specificity, and F1 score) heatmap of various feature selections and multiple machine learning classifiers belonging to the ‘Original Method’ implemented in the current study.

**Figure 9:**
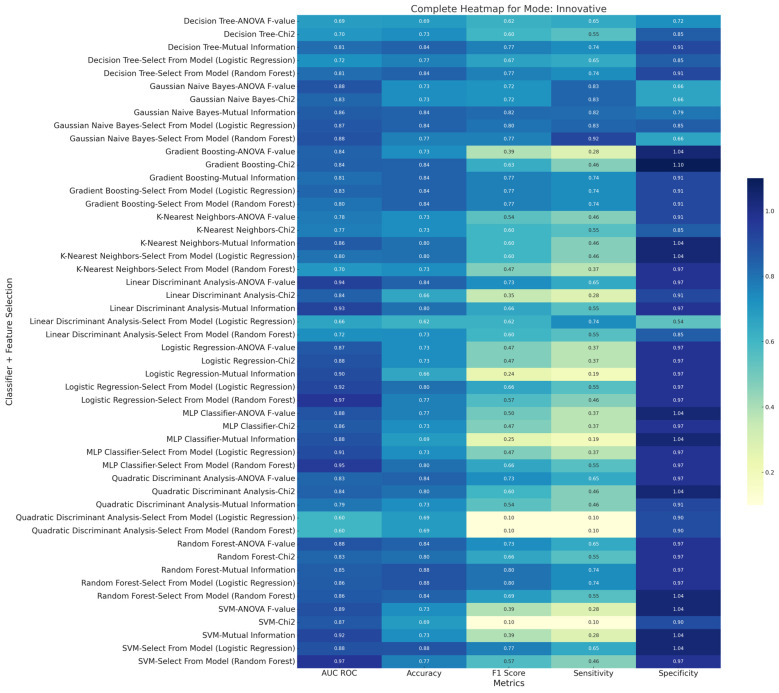
Diagnostic performance metric (AUC ROC, accuracy, sensitivity, specificity, and F1 score) heatmap of various feature selections and multiple machine learning classifiers belonging to the ‘Innovative Method’ implemented in the current study.

**Figure 10:**
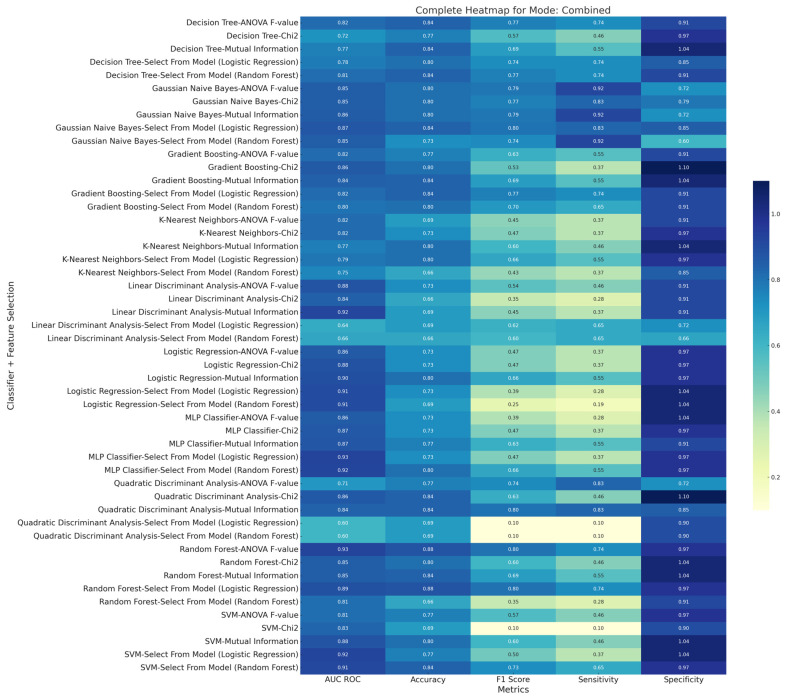
Diagnostic performance metric (AUC ROC, accuracy, sensitivity, specificity, and F1 score) heatmap of various feature selections and multiple machine learning classifiers belonging to the ‘Combined Method’ implemented in the current study.

Figure 8, Figure 9 and Figure 10 are the metric heatmaps of various feature selections and multiple classifiers implemented in this study. The innovative model representation evidently improved the classification performance, as seen through various metrics such as F1 score, accuracy, sensitivity, and specificity. The top three results across each of the three methods—original, innovative, and combined (original + innovative)—arranged by F1 scores are highlighted below:

### 3.1. Original Method

Classifier: Random Forest, Feature Selection: Select from Model (Logistic Regression), F1 Score: 0.7, Accuracy: 77.84%, AUC ROC: 77.77%, Sensitivity: 63.64%, Specificity: 87.5%.

Classifier: Decision Tree, Feature Selection: Mutual Information, F1 Score: 0.7, Accuracy: 77.27%, AUC ROC: 77.77%, Sensitivity: 63.63%, Specificity: 87.5%.

Classifier: Gradient Boosting, Feature Selection: Select from Model (Logistic Regression), F1 Score: 70.00%, Accuracy: 75.56%, AUC ROC: 77.77%, Sensitivity: 63.63%, Specificity: 87.5%.

### 3.2. Innovative Method

Classifier: Gaussian Naive Bayes, Feature Selection: Mutual Information, F1 Score: 0.82, Accuracy: 84.07%, AUC ROC: 85.56%, Sensitivity: 81.81%, Specificity: 78.75%.

Classifier: Random Forest, Feature Selection: Select from Model (Logistic Regression), F1 Score: 0.8, Accuracy: 87.78%, AUC ROC: 85.56%, Sensitivity: 73.64%, Specificity: 97.5%.

Classifier: Random Forest, Feature Selection: Mutual Information, F1 Score: 0.8, Accuracy: 87.78%, AUC ROC: 85%, Sensitivity: 73.64%, Specificity: 97.5%.

### 3.3. Combined (Original + Innovative) Method

Classifier: Random Forest, Feature Selection: ANOVA F-value, F1 Score: 0.8, Accuracy: 87.8%, AUC ROC: 92.67%, Sensitivity: 73.64%, Specificity: 97.5%.

Classifier: Random Forest, Feature Selection: Select from Model (Logistic Regression), F1 Score: 0.8, Accuracy: 87.78%, AUC ROC: 88.69%, Sensitivity: 73.64%, Specificity: 97.5%.

Classifier: Gaussian Naive Bayes, Feature Selection: Select from Model (Logistic Regression), F1 Score: 79.56%, Accuracy: 80.37%, AUC ROC: 86.98%, Sensitivity: 82.72%, Specificity: 85%.

The innovative and combined methods, with the incorporation of the novel model representation, notably outperformed the original method in terms of all metrics, showcasing the added value brought about by the innovative model representation.

The comparison between different machine learning classifiers and the feature selection algorithms implemented in this study are summarized in Supplemental Figures S1 and S2.

## 4. Discussion

The management of GBMs and BMs is vastly different and can potentially affect the clinical outcome. In general, the differentiation of these two neoplasms is possible based on the clinical history or presence of multiple enhancing lesions. However, distinction remains challenging when the patient presents with a solitary enhancing mass, as both of these neoplasms may exhibit ring enhancement and extensive vasogenic edema on conventional neuroimaging. Therefore, there is an unmet need to develop a prediction model for providing accurate and early differentiation of GBMs and BMs prior to the initiation of any therapeutic interventions. In the present study, we sought to develop a machine learning-based classification model to differentiate these two entities. Our findings suggest that quantitative physiological MRI parameters along with the feature engineering-guided creation of new features may be a promising machine learning approach for the accurate distinction of GBMs from BMs with high diagnostic performance (classification accuracy of 87.8%, area under the ROC curve of 92.67%, a sensitivity of 73.64%, and a specificity of 97.5%). This has great potential for prognostication and making appropriate therapeutic decisions in these patients.

Lately, several studies have reported the potential utility of machine learning-based radiomics features derived from anatomical images in discriminating GBMs from BMs with variable success [15,16,17,18,19,20]. In a study, Ning et al. [40] investigated seven machine learning-based classifiers and five feature reduction methods using radiomics features derived from post-contrast T1- and T2-weighted images and obtained an AUC of 0.89 and accuracy of 83%. Using texture features for differentiation between GBMs and BMs, some other studies have reported AUC results in the range between 0.68 and 0.96 [41,42,43,44,45]. Moving forward, a study demonstrated that dual-tree complex wavelet-transformed images had better diagnostic performance than pre-transformed and conventional wavelet-transformed images in discriminating these two entities [46]. 

In addition to machine learning-based algorithms, some other studies have used deep learning-based modules in distinguishing GBMs from BMs. Using the DenseNet121 model and multiparametric conventional neuroimaging in a study, Park et al. [47] were able to provide an external test classification of GBMs and SBMs with an AUC of 83.00%. The model incorporated the estimates of the predictive uncertainty, which proved to be more accurate and reliable in the low-uncertainty subgroups and provided an excellent detection of the out-of-distribution data, which improved clinical reliability. In another study, Liu et al. [48] used MFFC-Net, a deep learning classification network, for classifying brain neoplasms with an ACC of 0.92. These results outperformed a conventional radiomics model (ACC of 0.829). 

Despite these encouraging results, radiomics/deep learning-based features can vary considerably among different T1-weighted pulse sequences [49]. Moreover, those studies used high-dimensional shape/texture features such as grey-level co-occurrence matrix (GLCM), grey-level co-dependence matrix (GLDM), and grey-level run length matrix (GLRLM) extracted from anatomically and/or physiologically sensitive images (e.g., MD, CBV maps). These shape/texture-based features are highly sensitive to variations in image acquisition and processing protocols and are not associated with clinically meaningful interpretations related to tumor microenvironment and biology. In contrast, DTI- and DSC-PWI-derived parameters (e.g., MD, FA, CL, CBV) are widely used in clinical practice and provide direct physiologic linkage to tumor microenvironment and biology [7]. Using a Bayesian machine learning-based algorithm, a previous study developed a classification model derived from DTI- and DSC-PWI-derived parameters for differentiating GBMs from BMs with a predictive accuracy of 0.94 [50]. Although Bayesian networks are declarative, possess powerful inference capabilities, and can handle missing data, they are generally less popular than other machine learning-based models as Bayesian statistics require greater investigator expertise. Moreover, Bayesian network-based predictive models are computationally intensive and time-consuming, depend on prior chosen probabilities and assumptions that may not always hold true, and generally perform poorly on high-dimensional data [51]. Therefore, Bayesian network-based approaches may not always be feasible and useful in the real clinical workflow.

In the present study, FA from CERs and rCBV and MD from IPRs were identified as the highest-ranked predictive parameters among all independent features for distinguishing GBMs from BMs. The parameter FA signifies the degree of diffusion asymmetry present within a voxel and its value ranges from 0 (isotropic) to 1 (maximally anisotropic). It has been reported that organized microstructures secondary to closely packed proliferating tumor cells in brain neoplasms result in high FA [52]. Some previous studies have reported significantly higher FA from CER in GBMs than in BMs. This may be due to the directional orientation of the extracellular matrix in GBMs [53,54]. The extracellular space and matrix play an important role in tumor growth and infiltration. Neoplastic cells produce large amounts of tumor-specific extracellular matrix components, which serve as a substrate for adhesion and subsequent migration of the cells through the enlarged extracellular space [53]. These molecules accumulate and get oriented in extracellular matrix, resulting in high FA.

Importantly, we observed significant variations in MD and rCBV from IPRs. Our results and earlier published reports support the observation that the peritumoral region in GBMs is associated with the infiltration by neoplastic cells [55]. This invasive pattern has been attributed to the stem cell-like character of GBMs [56] and has been observed in both in vitro [57] and in vivo studies [58]. On the other hand, the IPR in BMs encompasses vasogenic edema with the presence of no or minimal neoplastic cells [59]. Therefore, probing the differences in imaging features from peritumor regions is an effective approach to differentiating GBMs from BMs. The significantly lower MD from the IPRs of GBMs observed in our study is consistent with previous observations. Lower MD is reflective of increased membrane turnover and cellular proliferation. The parameter rCBV is considered a potent biomarker to assess tumor angiogenesis and microvasculature in brain tumors [60]. Several studies [9,61,62,63,64] have reported that rCBV values from IPRs can differentiate GBMs from BMs based on degree of neoplastic infiltration in the peritumoral regions and quantitative assessment of neovascularization. Our findings in the current study are in agreement with these published results.

In the present study, a feature engineering method was used that leveraged the raw data to create a set of new features. Feature engineering offers several benefits, as this method helps in extracting more information and insights from raw data, mainly capturing interactions and relationships among features, revealing hidden patterns and trends in the data, and thereby increases the explanatory power of machine learning-based predictive models. Additionally, feature engineering improves the accuracy and robustness of predictive models by eliminating potential sources of inconsistencies by filling in the missing values, reducing the adverse effects of data overfitting, and nullifying the negative impact of outliers. However, feature engineering is also associated with several challenges as it requires deep technical skills and detailed knowledge of data engineering, and this method can be time-consuming, labor-intensive, and source-intensive. Additionally, feature engineering can be subjective and biased due to making assumptions and choices about features while dealing with trade-offs and uncertainties. Therefore, more such studies are required to confirm the potential utility of feature engineering approach in neurooncological applications. 

In the current study, 10 commonly used machine learning classifiers and five feature selection algorithms were used on 14 image features, including five from DTI (MD, FA, CL, CP, and CS), and two from DSC-PWI (rCBV and rCBV_max_) derived from both CER and IPR of neoplasms to construct prediction models. Classifiers were selected based on accuracy, training duration, handling of missing data, and ease of interpretation. Using conventional, innovative, and combined feature selection methods, we demonstrated that several classifiers performed well in distinguishing GBMs from BMs during training and testing with variable degrees of diagnostic metrics. However, the random forest classifier with an ANOVA F-value feature selection algorithm using the combined method showed the best diagnostic performance in distinguishing GBMs from single BMs with a classification accuracy of 87.8%. The random forest classifier is an ensemble method that uses a collection of hierarchical tree structures for decision making in classification and regression problems. Previous studies have shown the great potential of random forest classifiers in various clinical applications in brain tumors [65,66,67], including the differentiation of GBMs from BMs [17], which may due to the fact that random forest is less sensitive to bias and data-overfitting issues than any other binary classifiers. Our results also highlight the significance of using a combination of both conventional and engineered features for more effective classification of brain tumors, as this combination was able to capture greater degrees of intratumoral and peritumoral heterogeneities present within GBMs and BMs than those situations when these features were used in isolation.

While presenting with promising findings, our study had some shortcomings, including the retrospective nature of the study design. This was a single-center study with a small patient cohort, and no external validation of our findings was performed to further establish model generalizability. As deep learning-based algorithms typically require large-scale datasets to effectively train the numerous parameters without causing any selection bias, data overfitting, and spurious relationships, we used a classical machine learning approach in the present study. Additionally, due to the novelty of the feature engineering approach, future studies are necessary to confirm the efficacy and robustness of the feature engineering approach in various clinical applications. Another limitation was that a signal intensity thresholding-based semi-automatic segmentation method was used to delineate each lesion into CERs and IPRs in the present study. However, manual inspections were performed by an experienced, board-certified neuroradiologist to correct for any pixel anomalies present within the segmend regions. While semi-automatic segmentation has been successfully used by our group and others in neurooncological applications, advanced deep learning-based tumor segmentation techniques should be used in future studies to enhance the segmentation reliability.

It is well known that even small differences in hardware/software components or sequence parameters may result in substantial variations in image signal intensity and contrast patterns, hindering the correct interpretation of imaging results obtained from different MRI protocols, scanner vendors and treatment centers. Therefore, the widespread adoption of these imaging techniques into routine clinical workflow requires standardization, harmonization of data acquisition, and processing protocols, along with the application of well-defined quality assessment/control procedures. Fortunately, consensus guidelines have been proposed to implement MR diffusion, and perfusion imaging techniques across different clinical sites [68,69]. Additional improvements in this field require data sharing and conduction of large multicentric validation studies.

## 5. Conclusions

A combination of physiologically sensitive and quantitative MRI parameters along with our feature-engineered technique may create an accurate machine learning-based classification model in distinguishing GBMs from BMs. Our findings indicate that feature engineering has a positive impact on the model’s performance. The incorporation of both feature engineering-derived interaction features and non-interacting/conventional features together in the model’s development resulted in improved diagnostic performance (AUC = 92.67% vs. ~78–85%) in comparison to the situations when only conventional or interaction features were used independently in distinguishing GBMs from BMs.

## Figures and Tables

**Table 1:** Demographics of GBM and BM Patients.

Glioblastomas (*N* = 62)			Solitary Brain Metastases (*N* = 26)		
Characteristic		*N*	Characteristic		*N*
**Age (Y ± SD)**	59.2 ± 13.7		**Age (Y ± SD)**	57.1 ± 11.5	
**Gender**	Male	37	**Gender**	Male	14
	Female	25		Female	12
**Brain Location**	Temporal	25	**Brain Location**	Temporal	8
	Frontal	16		Frontal	9
	Parietal	7		Parietal	3
	Frontotemporal	4		Frontotemporal	2
	Temporoparietal	4		Temporoparietal	1
	Occipitoparietal	3		Frontoparietal	1
	Frontoparietal	2		Occipital	2
	Occipital	1	**Primary Site**	Lung	19
				Breast	5
				Melanoma	1
				Colon	1

## Data Availability

The data presented in this study are available on request from the corresponding author (SC or SAH).

## Supplementary Materials

The following supporting information can be downloaded at: https://www.mdpi.com/article/10.3390/diagnostics15010038/s1, Supplemental Figure S1: Area under the ROC curve distribution per feature selections based on three different methods (original, innovative, and combined). Supplemental Figure S2: Area under the ROC curve distribution per machine learning classifiers based on three different methods (original, innovative, and combined).
