# Supplementary material for: Quantitative Physiologic MRI Combined with Feature Engineering for Developing Machine Learning-Based Prediction Models to Distinguish Glioblastomas from Single Brain Metastases
Source: Diagnostics (Basel). 2024 Dec 27;15(1):38. doi: 10.3390/diagnostics15010038 (PMC11720653; doi:10.3390/diagnostics15010038)
Supplement: Supplementary file 1 [file diagnostics-15-00038-s001.zip › diagnostics-3308021-supplementary.pdf]

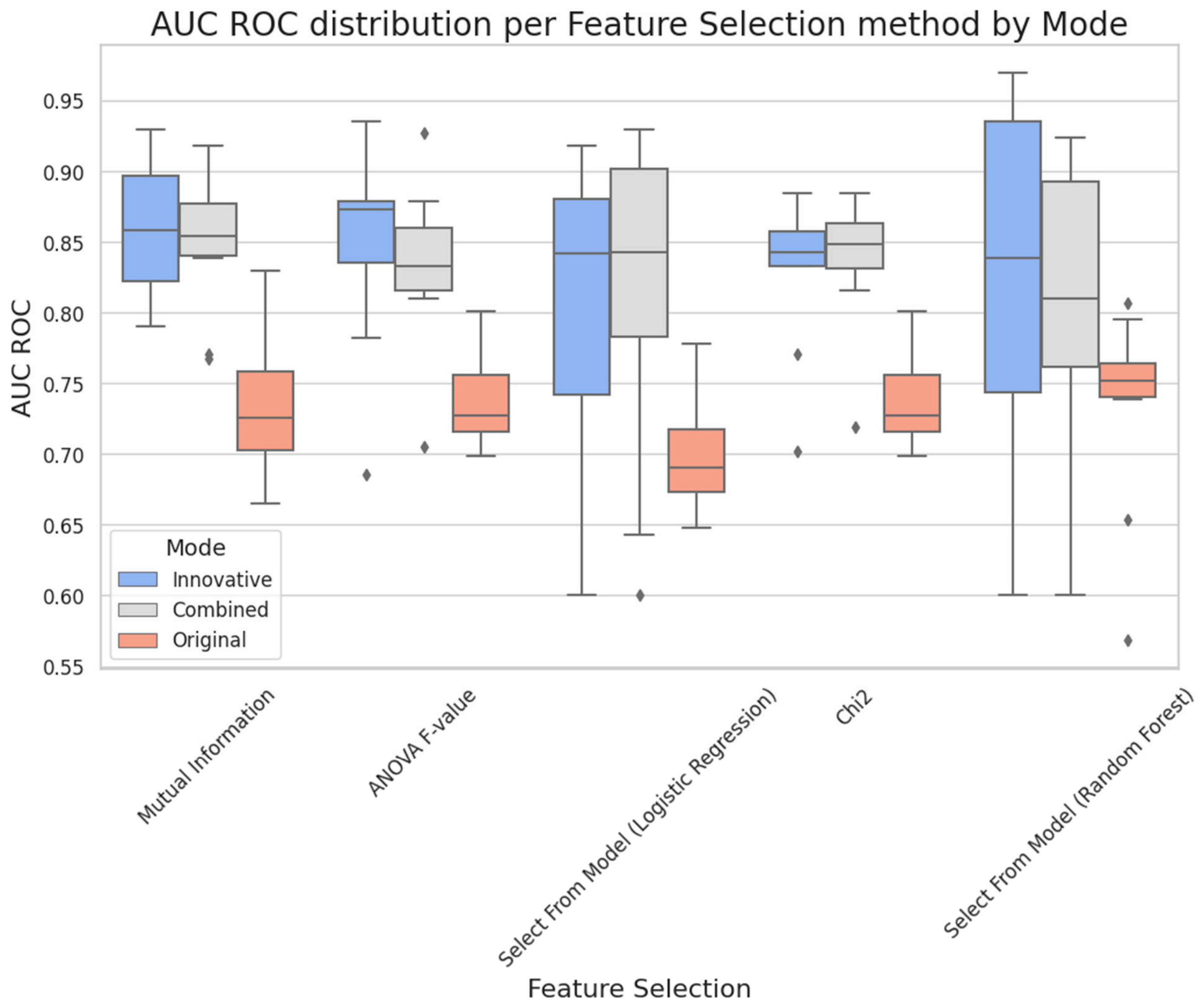


**Supplementary Figure S1.** Area under the ROC curve distribution per feature selections based on three different methods (original, innovative, and combined).

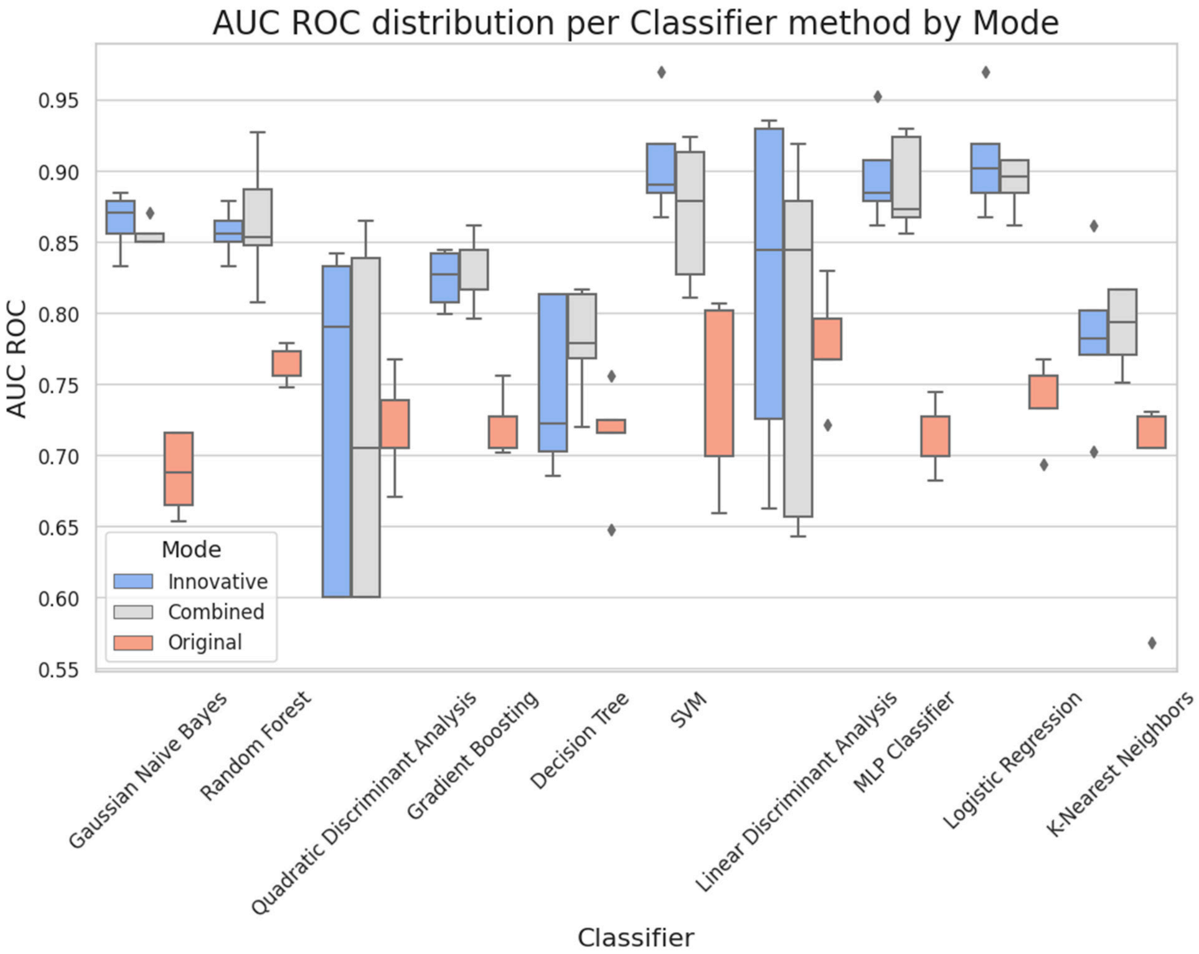


**Supplementary Figure S2.** Area under the ROC curve distribution per machine learning classifiers based on three different methods (original, innovative, and combined).
